# (Acetone-κ*O*){6,6′-di-*tert*-butyl-2,2′-[1,2-phenyl­enebis(nitrilo­methyl­idyne)]diphenolato-κ^4^ *O*,*N*,*N*′,*O*′}zinc(II)

**DOI:** 10.1107/S1600536808011215

**Published:** 2008-04-30

**Authors:** Naser Eltaher Eltayeb, Siang Guan Teoh, Suchada Chantrapromma, Hoong-Kun Fun, Rohana Adnan

**Affiliations:** a School of Chemical Science, Universiti Sains Malaysia, 11800 USM, Penang, Malaysia; b Department of Chemistry, Faculty of Science, Prince of Songkla University, Hat-Yai, Songkhla 90112, Thailand; c X-ray Crystallography Unit, School of Physics, Universiti Sains Malaysia, 11800 USM, Penang, Malaysia

## Abstract

The mol­ecule of the title compound, [Zn(C_28_H_30_N_2_O_2_)(CH_3_COCH_3_)], lies across a mirror plane with the Zn^II^ ion and the acetone mol­ecule on the mirror plane. The Zn^II^ ion is in a five-coordinate distorted square-pyramidal N_2_O_3_ environment, with the two imine N and two phenolic O atoms of the tetra­dentate Schiff base dianion in the basal plane and the acetone mol­ecule in the apical position. The central benzene ring makes a dihedral angle of 16.5 (2)° with the two outer phenolate rings. In the crystal structure, the mol­ecules are arranged into anti­parallel columns along the *a* axis.

## Related literature

For bond-length data, see: Allen *et al.* (1987 ▶). For related structures, see: Eltayeb *et al.* (2007*a* ▶,*b* ▶,*c* ▶); Reglinski *et al.* (2002 ▶). For background to the applications of zinc complexes, see, for example: Assaf & Chung (1984 ▶); Basak *et al.* (2007 ▶); Berg & Shi (1996 ▶); Tarafder *et al.* (2002 ▶).
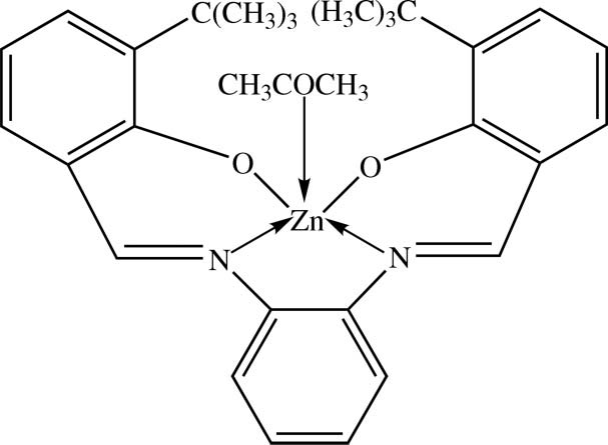


## Experimental

### 

#### Crystal data

[Zn(C_28_H_30_N_2_O_2_)(C_3_H_6_O)]
*M* *_r_* = 550.11
Monoclinic, 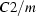

*a* = 10.5803 (16) Å
*b* = 16.3602 (19) Å
*c* = 15.729 (2) Å
β = 94.446 (10)°
*V* = 2714.4 (6) Å^3^
*Z* = 4
Mo *K*α radiation
μ = 0.94 mm^−1^
*T* = 100.0 (1) K
0.57 × 0.24 × 0.07 mm

#### Data collection

Bruker SMART APEXII CCD area-detector diffractometer
Absorption correction: multi-scan (*SADABS*; Bruker, 2005 ▶) *T*_min_ = 0.616, *T*_max_ = 0.935
29567 measured reflections
2756 independent reflections
2613 reflections with *I* > 2σ(*I*)
*R*_int_ = 0.089

#### Refinement

*R*[*F* ^2^ > 2σ(*F* ^2^)] = 0.073
*wR*(*F* ^2^) = 0.190
*S* = 1.20
2756 reflections
178 parameters
H-atom parameters constrained
Δρ_max_ = 1.50 e Å^−3^
Δρ_min_ = −1.15 e Å^−3^

### 

Data collection: *APEX2* (Bruker, 2005 ▶); cell refinement: *APEX2*; data reduction: *SAINT* (Bruker, 2005 ▶); program(s) used to solve structure: *SHELXTL* (Sheldrick, 2008 ▶); program(s) used to refine structure: *SHELXTL*; molecular graphics: *SHELXTL*; software used to prepare material for publication: *SHELXTL* and *PLATON* (Spek, 2003 ▶).

## Supplementary Material

Crystal structure: contains datablocks global, I. DOI: 10.1107/S1600536808011215/ci2585sup1.cif

Structure factors: contains datablocks I. DOI: 10.1107/S1600536808011215/ci2585Isup2.hkl

Additional supplementary materials:  crystallographic information; 3D view; checkCIF report

## Figures and Tables

**Table 1:** Hydrogen-bond geometry (Å, °)

*D*—H⋯*A*	*D*—H	H⋯*A*	*D*⋯*A*	*D*—H⋯*A*
C13—H13*B*⋯O1	0.96	2.37	3.022 (7)	124
C14—H14*C*⋯O1	0.96	2.41	2.983 (6)	118

**Fig. 1.**
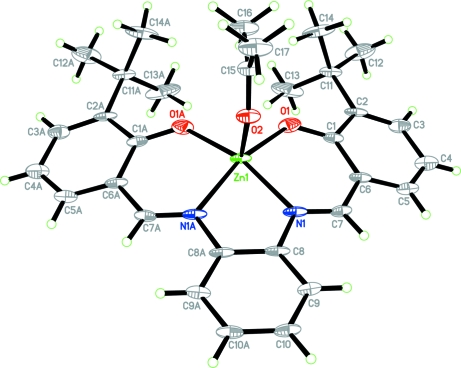
The structure of the title complex, showing 50% probability displacement ellipsoids and the atomic numbering. Atoms labeled with the suffix A are generated by the symmetry operation (x, -y, z). Atoms Zn1, O2, C15, C16 and C17 lie on the crystallographic mirror plane.

**Fig. 2.**
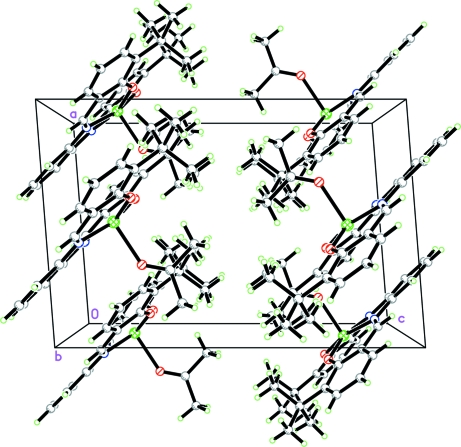
The crystal packing of the title compound, viewed approximately along the b axis.

## supplementary crystallographic information

### Comment

Schiff base ligands containing oxygen and imine nitrogen atoms and their metal complexes have gained increased interest in the field of synthetic chemistry due to their variety of applications. Zinc complexes play important roles in various biological systems such as neurotransmission, signal transduction and gene expression (Assaf & Chung, 1984; Berg & Shi, 1996). It is well known that certain zinc complexes with Schiff-bases are biologically active and show very good cytotoxicity against leukemic cells (Tarafder *et al.*, 2002). Previously, we reported the crystal structures of five coordination Zn^II^ complexes with Schiff base ligands (Eltayeb *et al.*, 2007*a*;2007*b*; 2007*c*. As a continuation of our research on Schiff base complexes, we report here the crystal structure of a Zn^II^ complex of a closely-related ligand.

The asymmetric unit of the title compound contain one-half of the [Zn(C_28_H_30_N_2_O_2_)(CH_3_COCH_3_)] complex, with the other half generated by a crystallographic mirror plane. Atoms Zn1, O2, C15, C16, C17, H16A and H17A lie on the mirror plane. The Zn^II^ ion is five-coordinate and adopts a distorted square-pyramidal geometry, with the two imine N (N1 and N1A) and two phenolic O (O1 and O1A) atoms of the tetradentate Schiff base dianion forming a square base, while the acetone molecule occupies the apical coordination site. The two phenolic O atoms and two imine N atoms are in mutually *cis* positions. The Zn—O and Zn—N distances in the N_2_O_2_ coordination plane [1.949 (4) and 2.078 (4) Å, respectively] are in the same ranges as those observed in the other closely related Zn^II^ complexes of N_2_O_2_ Schiff base ligands (Eltayeb *et al.*, 2007*a*; 2007*b*; 2007*c*; Reglinski *et al.* (2002). The apical Zn—O(acetone) distance is 2.182 (4) Å. Other bond lengths and angles observed in the structure are also normal (Allen *et al.*, 1987). The Zn^II^ ion is displaced from the O1/N1/O1A/N1A plane by 0.023 Å toward the apical acetone molecule. The basal bond angles O–Zn–N [O1–Zn1–N1 = 90.24 (15) °] are close to 90° whereas the O–Zn–O [O1–Zn1–O1A = 97.4 (2)°] angle is bigger than 90° and the N–Mn–N [N1–Zn1–N1A = 78.9 (2)°] angle is smaller than 90°. The bond angles between the O2 atom of the acetone molecule and the atoms in the basal plane are in the range 91.02 (13) to 101.67 (13)°, indicating a distorted square-pyramidal geometry. Coordination of the the N_2_O_2_ chelate ligand to the Zn^II^ ion results in the formation of a five-membered ring (Zn1/N1/C8/C8A/N1A) and two six-membered rings *viz*. Zn1/O1/C1/C6/C7/N1 and Zn1/O1A/C1A/C6A/C7A/N1A. The central benzene ring (C8–C9–C10–C8A–C9A–C10A) makes a dihedral angle of 16.5 (2)° with the outer phenolate rings.

Intramolecular C—H···O weak interactions are observed (Table 1). In the crystal packing (Fig. 2), the molecules are arranged into antiparallel columns along the *a* axis.

### Experimental

The title compound was synthesized by adding 3-*tert*-butyl-2-hydroxybenzaldehyde (0.7 ml, 4 mmol) to a solution of *o*-phenylenediamine (0.216 g, 2 mmol) in ethanol 95% (20 ml). The mixture was refluxed with stirring for half an hour. Zinc chloride (0.272 g, 2 mmol) in ethanol (10 ml) was then added, followed by triethylamine (0.5 ml, 3.6 mmol). The mixture was refluxed at room temperature for two hours. A yellow-orange precipitate was obtained. Yellow single crystals of the title compound suitable for X-ray structure determination were recrystallized from acetone by slow evaporation of the solvent at room temperature after a few days.

### Refinement

All H atoms were placed in calculated positions with d(C—H) = 0.93 Å, *U*_iso_ = 1.2*U*_eq_(C) for aromatic and 0.96 Å, *U*_iso_ = 1.5*U*_eq_(C) for CH_3_ atoms. A rotating group model was used for the methyl groups. The highest residual electron density peak is located at 1.03 Å from Zn1 and the deepest hole is located at 0.84 Å from Zn1.

### Crystal data

**Table:**

[Zn(C_28_H_30_N_2_O_2_)(C_3_H_6_O)]	*F*_000_ = 1160
*M**_r_* = 550.11	*D*_x_ = 1.346 Mg m^−^^3^
Monoclinic, *C*2/*m*	Mo *K*α radiation λ = 0.71073 Å
Hall symbol: -C 2y	Cell parameters from 2756 reflections
*a* = 10.5803 (16) Å	θ = 2.3–26.0º
*b* = 16.3602 (19) Å	µ = 0.94 mm^−^^1^
*c* = 15.729 (2) Å	*T* = 100.0 (1) K
β = 94.446 (10)º	Plate, yellow
*V* = 2714.4 (6) Å^3^	0.57 × 0.24 × 0.07 mm
*Z* = 4	

### Data collection

**Table:**

Bruker SMART APEXII CCD area-detector diffractometer	2756 independent reflections
Radiation source: fine-focus sealed tube	2613 reflections with *I* > 2σ(*I*)
Monochromator: graphite	*R*_int_ = 0.090
Detector resolution: 8.33 pixels mm^-1^	θ_max_ = 26.0º
*T* = 100.0(1) K	θ_min_ = 2.3º
ω scans	*h* = −13→13
Absorption correction: multi-scan(SADABS; Bruker, 2005)	*k* = −20→20
*T*_min_ = 0.616, *T*_max_ = 0.935	*l* = −19→19
29567 measured reflections	

### Refinement

**Table:**

Refinement on *F*^2^	Secondary atom site location: difference Fourier map
Least-squares matrix: full	Hydrogen site location: inferred from neighbouring sites
*R*[*F*^2^ > 2σ(*F*^2^)] = 0.073	H-atom parameters constrained
*wR*(*F*^2^) = 0.190	*w* = 1/[σ^2^(*F*_o_^2^) + (0.0669*P*)^2^ + 29.8359*P*] where *P* = (*F*_o_^2^ + 2*F*_c_^2^)/3
*S* = 1.20	(Δ/σ)_max_ = 0.001
2756 reflections	Δρ_max_ = 1.50 e Å^−^^3^
178 parameters	Δρ_min_ = −1.15 e Å^−^^3^
Primary atom site location: structure-invariant direct methods	Extinction correction: none

### Special details

**Table:**

Experimental. The low-temperature data was collected with the Oxford Cyrosystem Cobra low-temperature attachment.
Geometry. All e.s.d.'s (except the e.s.d. in the dihedral angle between two l.s. planes) are estimated using the full covariance matrix. The cell e.s.d.'s are taken into account individually in the estimation of e.s.d.'s in distances, angles and torsion angles; correlations between e.s.d.'s in cell parameters are only used when they are defined by crystal symmetry. An approximate (isotropic) treatment of cell e.s.d.'s is used for estimating e.s.d.'s involving l.s. planes.
Refinement. Refinement of *F*^2^ against ALL reflections. The weighted *R*-factor *wR* and goodness of fit *S* are based on *F*^2^, conventional *R*-factors *R* are based on *F*, with *F* set to zero for negative *F*^2^. The threshold expression of *F*^2^ > σ(*F*^2^) is used only for calculating *R*-factors(gt) *etc*. and is not relevant to the choice of reflections for refinement. *R*-factors based on *F*^2^ are statistically about twice as large as those based on *F*, and *R*- factors based on ALL data will be even larger.

### Fractional atomic coordinates and isotropic or equivalent isotropic displacement parameters (Å^2^)

**Table:**

	*x*	*y*	*z*	*U*_iso_*/*U*_eq_	
Zn1	0.50605 (6)	0.0000	0.18490 (4)	0.0170 (3)	
O1	0.6013 (3)	0.0895 (2)	0.2409 (2)	0.0271 (8)	
O2	0.3336 (4)	0.0000	0.2526 (3)	0.0231 (11)	
N1	0.4250 (3)	0.0807 (3)	0.0944 (2)	0.0208 (9)	
C1	0.6146 (4)	0.1649 (3)	0.2177 (3)	0.0185 (10)	
C2	0.7084 (4)	0.2160 (3)	0.2638 (3)	0.0213 (10)	
C3	0.7224 (5)	0.2950 (3)	0.2378 (3)	0.0264 (11)	
H3A	0.7845	0.3269	0.2666	0.032*	
C4	0.6474 (5)	0.3306 (4)	0.1694 (3)	0.0316 (12)	
H4A	0.6589	0.3849	0.1543	0.038*	
C5	0.5578 (4)	0.2836 (3)	0.1261 (3)	0.0251 (11)	
H5A	0.5069	0.3064	0.0814	0.030*	
C6	0.5411 (4)	0.2014 (3)	0.1477 (3)	0.0197 (10)	
C7	0.4485 (4)	0.1576 (3)	0.0931 (3)	0.0186 (10)	
H7A	0.4008	0.1884	0.0526	0.022*	
C8	0.3366 (4)	0.0429 (3)	0.0341 (3)	0.0206 (10)	
C9	0.2534 (4)	0.0848 (4)	−0.0238 (3)	0.0227 (10)	
H9A	0.2520	0.1417	−0.0234	0.027*	
C10	0.1733 (4)	0.0427 (4)	−0.0815 (3)	0.0260 (11)	
H10A	0.1171	0.0717	−0.1217	0.031*	
C11	0.7916 (4)	0.1815 (3)	0.3403 (3)	0.0230 (11)	
C12	0.8813 (6)	0.2459 (5)	0.3807 (4)	0.0481 (18)	
H12A	0.8329	0.2907	0.4005	0.072*	
H12B	0.9361	0.2653	0.3392	0.072*	
H12C	0.9314	0.2223	0.4279	0.072*	
C13	0.8731 (6)	0.1118 (5)	0.3111 (4)	0.057 (2)	
H13A	0.9317	0.1325	0.2727	0.086*	
H13B	0.8198	0.0712	0.2825	0.086*	
H13C	0.9192	0.0877	0.3598	0.086*	
C14	0.7089 (5)	0.1515 (5)	0.4096 (3)	0.0411 (17)	
H14A	0.6484	0.1930	0.4212	0.062*	
H14B	0.7614	0.1399	0.4607	0.062*	
H14C	0.6649	0.1027	0.3905	0.062*	
C15	0.3125 (6)	0.0000	0.3276 (4)	0.0202 (14)	
C16	0.4167 (6)	0.0000	0.3977 (4)	0.0337 (19)	
H16A	0.4975	0.0000	0.3738	0.051*	
H16B	0.4088	−0.0479	0.4325	0.051*	
C17	0.1792 (7)	0.0000	0.3533 (5)	0.037 (2)	
H17A	0.1213	0.0000	0.3031	0.055*	
H17B	0.1646	−0.0479	0.3868	0.055*	

### Atomic displacement parameters (Å^2^)

**Table:**

	*U*^11^	*U*^22^	*U*^33^	*U*^12^	*U*^13^	*U*^23^
Zn1	0.0076 (4)	0.0354 (5)	0.0077 (4)	0.000	−0.0021 (2)	0.000
O1	0.0237 (17)	0.040 (2)	0.0163 (16)	−0.0073 (15)	−0.0093 (13)	0.0022 (15)
O2	0.013 (2)	0.041 (3)	0.015 (2)	0.000	0.0024 (17)	0.000
N1	0.0078 (16)	0.047 (3)	0.0077 (16)	−0.0002 (17)	−0.0016 (13)	−0.0003 (16)
C1	0.0103 (19)	0.035 (3)	0.0099 (19)	0.0010 (18)	0.0022 (15)	−0.0028 (18)
C2	0.0092 (19)	0.048 (3)	0.0070 (19)	−0.004 (2)	0.0026 (15)	−0.0035 (19)
C3	0.022 (2)	0.041 (3)	0.017 (2)	−0.010 (2)	−0.0006 (18)	−0.001 (2)
C4	0.032 (3)	0.045 (3)	0.018 (2)	−0.010 (2)	0.000 (2)	0.001 (2)
C5	0.022 (2)	0.042 (3)	0.012 (2)	0.000 (2)	0.0012 (17)	0.002 (2)
C6	0.0091 (19)	0.042 (3)	0.0086 (19)	−0.0013 (19)	0.0027 (15)	−0.0027 (19)
C7	0.0098 (19)	0.036 (3)	0.010 (2)	0.0016 (18)	−0.0002 (15)	0.0029 (18)
C8	0.0071 (18)	0.048 (3)	0.0069 (18)	0.0005 (19)	0.0013 (15)	−0.0008 (18)
C9	0.015 (2)	0.041 (3)	0.012 (2)	0.000 (2)	0.0003 (16)	0.0039 (19)
C10	0.015 (2)	0.050 (3)	0.012 (2)	0.004 (2)	−0.0046 (17)	0.002 (2)
C11	0.012 (2)	0.047 (3)	0.010 (2)	−0.002 (2)	0.0007 (16)	−0.002 (2)
C12	0.039 (3)	0.079 (5)	0.023 (3)	−0.027 (3)	−0.017 (2)	0.010 (3)
C13	0.037 (3)	0.112 (7)	0.021 (3)	0.041 (4)	−0.015 (2)	−0.018 (3)
C14	0.015 (2)	0.094 (5)	0.014 (2)	−0.010 (3)	−0.0021 (18)	0.015 (3)
C15	0.009 (3)	0.036 (4)	0.016 (3)	0.000	0.002 (2)	0.000
C16	0.016 (3)	0.069 (6)	0.016 (3)	0.000	−0.001 (3)	0.000
C17	0.015 (3)	0.075 (6)	0.021 (4)	0.000	0.007 (3)	0.000

### Geometric parameters (Å, °)

**Table:**

Zn1—O1^i^	1.949 (4)	C9—C10	1.378 (7)
Zn1—O1	1.949 (4)	C9—H9A	0.93
Zn1—N1	2.078 (4)	C10—C10^i^	1.396 (12)
Zn1—N1^i^	2.078 (4)	C10—H10A	0.96
Zn1—O2	2.182 (4)	C11—C13	1.522 (8)
O1—C1	1.296 (6)	C11—C12	1.523 (8)
O2—C15	1.218 (8)	C11—C14	1.532 (6)
N1—C7	1.283 (7)	C12—H12A	0.96
N1—C8	1.420 (6)	C12—H12B	0.96
C1—C6	1.430 (6)	C12—H12C	0.96
C1—C2	1.449 (6)	C13—H13A	0.96
C2—C3	1.367 (8)	C13—H13B	0.96
C2—C11	1.541 (6)	C13—H13C	0.96
C3—C4	1.412 (7)	C14—H14A	0.96
C3—H3A	0.93	C14—H14B	0.96
C4—C5	1.362 (7)	C14—H14C	0.96
C4—H4A	0.93	C15—C17	1.497 (9)
C5—C6	1.401 (8)	C15—C16	1.498 (9)
C5—H5A	0.93	C16—H16A	0.96
C6—C7	1.442 (6)	C16—H16B	0.96
C7—H7A	0.93	C17—H17A	0.96
C8—C9	1.396 (6)	C17—H17B	0.96
C8—C8^i^	1.404 (11)		
			
O1^i^—Zn1—O1	97.4 (2)	C10—C9—C8	120.5 (5)
O1^i^—Zn1—N1	163.48 (15)	C10—C9—H9A	119.7
O1—Zn1—N1	90.24 (15)	C8—C9—H9A	119.7
O1^i^—Zn1—N1^i^	90.23 (15)	C9—C10—C10^i^	120.0 (3)
O1—Zn1—N1^i^	163.48 (15)	C9—C10—H10A	120.3
N1—Zn1—N1^i^	78.9 (2)	C10^i^—C10—H10A	119.7
O1^i^—Zn1—O2	101.67 (13)	C13—C11—C12	107.2 (5)
O1—Zn1—O2	101.67 (13)	C13—C11—C14	110.0 (6)
N1—Zn1—O2	91.02 (13)	C12—C11—C14	107.2 (4)
N1^i^—Zn1—O2	91.02 (13)	C13—C11—C2	110.0 (4)
C1—O1—Zn1	130.6 (3)	C12—C11—C2	111.9 (5)
C15—O2—Zn1	134.1 (4)	C14—C11—C2	110.5 (4)
C7—N1—C8	122.3 (4)	C11—C12—H12A	109.5
C7—N1—Zn1	124.3 (3)	C11—C12—H12B	109.5
C8—N1—Zn1	113.4 (3)	H12A—C12—H12B	109.5
O1—C1—C6	123.4 (4)	C11—C12—H12C	109.5
O1—C1—C2	119.6 (4)	H12A—C12—H12C	109.5
C6—C1—C2	117.0 (4)	H12B—C12—H12C	109.5
C3—C2—C1	118.8 (4)	C11—C13—H13A	109.5
C3—C2—C11	120.7 (4)	C11—C13—H13B	109.5
C1—C2—C11	120.4 (5)	H13A—C13—H13B	109.5
C2—C3—C4	123.5 (5)	C11—C13—H13C	109.5
C2—C3—H3A	118.3	H13A—C13—H13C	109.5
C4—C3—H3A	118.3	H13B—C13—H13C	109.5
C5—C4—C3	118.4 (5)	C11—C14—H14A	109.5
C5—C4—H4A	120.8	C11—C14—H14B	109.5
C3—C4—H4A	120.8	H14A—C14—H14B	109.5
C4—C5—C6	121.2 (5)	C11—C14—H14C	109.5
C4—C5—H5A	119.4	H14A—C14—H14C	109.5
C6—C5—H5A	119.4	H14B—C14—H14C	109.5
C5—C6—C1	121.1 (4)	O2—C15—C17	120.6 (6)
C5—C6—C7	115.2 (4)	O2—C15—C16	122.2 (6)
C1—C6—C7	123.7 (5)	C17—C15—C16	117.2 (6)
N1—C7—C6	127.0 (4)	C15—C16—H16A	109.8
N1—C7—H7A	116.5	C15—C16—H16B	109.1
C6—C7—H7A	116.5	H16A—C16—H16B	110.0
C9—C8—C8^i^	119.4 (3)	C15—C17—H17A	109.4
C9—C8—N1	124.8 (5)	C15—C17—H17B	110.0
C8^i^—C8—N1	115.8 (3)	H17A—C17—H17B	109.4
			
O1^i^—Zn1—O1—C1	157.2 (3)	C4—C5—C6—C1	2.1 (7)
N1—Zn1—O1—C1	−8.1 (4)	C4—C5—C6—C7	−176.2 (4)
N1^i^—Zn1—O1—C1	40.3 (8)	O1—C1—C6—C5	179.0 (4)
O2—Zn1—O1—C1	−99.2 (4)	C2—C1—C6—C5	−1.3 (6)
O1^i^—Zn1—O2—C15	50.10 (11)	O1—C1—C6—C7	−2.9 (7)
O1—Zn1—O2—C15	−50.10 (11)	C2—C1—C6—C7	176.8 (4)
N1—Zn1—O2—C15	−140.55 (12)	C8—N1—C7—C6	−176.1 (4)
N1^i^—Zn1—O2—C15	140.55 (12)	Zn1—N1—C7—C6	5.7 (6)
O1^i^—Zn1—N1—C7	−117.8 (6)	C5—C6—C7—N1	172.7 (4)
O1—Zn1—N1—C7	0.1 (4)	C1—C6—C7—N1	−5.5 (7)
N1^i^—Zn1—N1—C7	−167.4 (3)	C7—N1—C8—C9	−10.0 (6)
O2—Zn1—N1—C7	101.8 (4)	Zn1—N1—C8—C9	168.4 (3)
O1^i^—Zn1—N1—C8	63.9 (6)	C7—N1—C8—C8^i^	169.4 (3)
O1—Zn1—N1—C8	−178.2 (3)	Zn1—N1—C8—C8^i^	−12.2 (3)
N1^i^—Zn1—N1—C8	14.3 (3)	C8^i^—C8—C9—C10	−1.1 (5)
O2—Zn1—N1—C8	−76.6 (3)	N1—C8—C9—C10	178.3 (4)
Zn1—O1—C1—C6	10.3 (6)	C8—C9—C10—C10^i^	1.1 (5)
Zn1—O1—C1—C2	−169.4 (3)	C3—C2—C11—C13	−117.0 (6)
O1—C1—C2—C3	179.1 (4)	C1—C2—C11—C13	62.5 (6)
C6—C1—C2—C3	−0.6 (6)	C3—C2—C11—C12	2.0 (6)
O1—C1—C2—C11	−0.4 (6)	C1—C2—C11—C12	−178.6 (4)
C6—C1—C2—C11	179.9 (4)	C3—C2—C11—C14	121.3 (5)
C1—C2—C3—C4	1.9 (7)	C1—C2—C11—C14	−59.2 (6)
C11—C2—C3—C4	−178.6 (4)	Zn1—O2—C15—C17	180.0
C2—C3—C4—C5	−1.1 (8)	Zn1—O2—C15—C16	0.000 (1)
C3—C4—C5—C6	−0.9 (7)		

Symmetry codes: (i) *x*, −*y*, *z*.

### Hydrogen-bond geometry (Å, °)

**Table:**

*D*—H···*A*	*D*—H	H···*A*	*D*···*A*	*D*—H···*A*
C13—H13B···O1	0.96	2.37	3.022 (7)	124
C14—H14C···O1	0.96	2.41	2.983 (6)	118
